# Melbourne Notes

**Published:** 1909-10-23

**Authors:** 


### MELBOURNE NOTES.

### HEALTH ADMINISTRATION.

The applications of five gentlemen for the position of Chairman of the Board of Public Health dealt with by the State Cabinet which holds the power of appointment, resulted in the choice of Dr. B. Burnett Ham. Dr. Ham is the son of an old and wealthy colonist of Ballarat, Mr. David Ham, and is forty-four years of age. In 1900 he was appointed Commissioner of Public Health for the State of Queensland, where he organised the Department of Public Health, and now, as head, he holds administrative authority over 140 local governing bodies. He has published several medical works, and is regarded as specially skilful in the administration of Pure Food and Health Acts. His translation to Melbourne gives him no increase of emolument.

The (State) Government have in view several changes in the administration of the Public Health Department. The present Board is to be superseded by an Advisory Board—as in Queensland—with the new chairman as Health Commissioner. The Board is to consist of five members, exclusive of the Commissioner, who is, *ex-officio*, member and chairman. At least two of the five must be medical practitioners, and at least one of the remaining three must have had not less than two years' experience of local government as member of a local body. The Board will advise the Minister of Public Health on matters relating to the administration of the Health Act. The Board will be called together by the Minister when required, and the members will receive such salaries and allowances as the Governor in Council decides. The present Board has sometimes had slight friction with the Minister, and when it was reported last month that the General Post Office was in an insanitary condition the discovery was made that the Board of Public Health had no power to deal with public buildings.

### FEDERAL QUARANTINE.

It is not to be supposed that the quarantine affairs of a whole continent with some 8,000 miles of coast can be regulated with one stroke of the pen. Dr. Norris, Director of Federal Quarantine, is proceeding carefully, but already certain articles likely to act as "fomites" are prohibited from entering the Commonwealth. Such are rags, second-hand bedding, second-hand clothing from any proclaimed place, flock, and bedding or articles stuffed or packed with flock, human bones (other than specimens for scientific purposes), human hair, jute bags, second-hand carpets and similar articles from proclaimed places to be admitted after disinfection carried out to the satisfaction of a quarantine officer. Some delay has occurred in the examination and clearing of the first mail steamers arriving under Federal Quarantine Regulations, which came into operation on August 1, but as the general standard of inspection is raised all round the coast the inspection at first port of call will suffice. Vessels arriving between the hours of 5 A.M and midnight will be inspected at once, mail steamers at whatever hour they reach port. Dr. Norris will shortly start on a tour of all quarantine stations, settled or proposed.

